# Suboptimal control of lipid levels: results from the non-interventional Centralized Pan-Russian Survey of the Undertreatment of Hypercholesterolemia II (CEPHEUS II)

**DOI:** 10.1186/s12933-017-0641-4

**Published:** 2017-12-16

**Authors:** Sergey Boytsov, Natalia Logunova, Yunona Khomitskaya, on behalf of the CEPHEUS II investigators, Eradzh Nuraliev, Anastasiya Lebedeva, Inessa Shchelkunova, Elena Vershuta, Svetlana Zhidkova, Veronika Rostorotskaya, Yana Shadaniya, Andrey Ivanov, Irina Zobenko, Olga Kvasova, Svetlana Zikun, Alina Glushchenya, Alexander Rumyantsev, Svetlana Prokof’eva, Mariya Baturova, Rodion Oseshnyk, Elena Zhukova, Irina Shumikhina, Vera Eltisheva, Larisa Bugaets, Vladimir Chernysh, Marina Ivochkina, Lyudmila Svistunova, Natalia Klimenko, Olga Kulchitskaya, Valentina Alexandrova, Marina Stepanova, Tatiana Chernysh, Galina Ivanchura, Olga Chachshina, Elena Afonina, Andrey Chernyshev, Evgeniya Korostyleva, Tatyana Staroverova, Yuriy Badin, Galina Il’icheva, Lyudmila Monetkina, Oxana Novikova, Olga Abashina, Galina Plaksina, Svetlana Kostomarova, Ekaterina Alieva, Olga Budanova, Inessa Kartashova, Svetlana Chepurnenko, Elena Oreshina, Natalia Skachkova, Rkiya Khanbekova, Olga Pashchenko, Yuliya Zolotova, Elena Volodina, Konstantin Fisher, Anastasiya Shurkevich, Elena Vikhman, Irina Poshinova, Vladislav Abramov, Daniil Cherepnin, Olga Leonova, Olga Kargina, Galina Gerent, Natalia Nabokikh, Galina Sokolovskikh, Valeria Tkhorikova, Lyudmila Titova, Svetlana Rachkova, Andrey Baglikov, Marinsa Giorgadze, Alexander Malygin, Svetlana Strelkova, Tatyana Ryzhova, Nina Kochladze, Natalia Vyasova, Sergey Vasiliev, Inna Bondarenko, Elena Mokhnacheva, Lyudmila Shumilina, Svetlana Pakhomova

**Affiliations:** 1 The Russian Cardiology Research and Production Complex, Moscow, Russian Federation; 2 Medical Affairs, AstraZeneca, Moscow, Russian Federation

**Keywords:** Cardiovascular disease, Hypercholesterolemia, Lipid-lowering drugs, Statins, Low-density lipoprotein cholesterol, Diabetes mellitus, Treatment targets

## Abstract

**Background:**

Elevated levels of low-density lipoprotein cholesterol (LDL-C) and glycosylated hemoglobin (HbA1c) are risk factors for cardiovascular complications. This study evaluated LDL-C goal attainment in Russian clinical practice among patients with moderate to very high cardiovascular risk. The study also assessed LDL-C goal attainment in patients prescribed lipid-lowering therapy for primary compared with secondary cardiovascular disease (CVD) prevention, predictors of LDL-C goal attainment, and the proportion of individuals with diabetes mellitus who achieved HbA1c < 7%.

**Methods:**

The Centralized Pan-Russian Survey on the Undertreatment of Hypercholesterolemia in Russia II (CEPHEUS II) was a multicenter, non-interventional, cross-sectional study conducted in the Russian Federation from September 2014 to November 2015. Participants were aged ≥ 18 years, were receiving a stable dose of lipid-lowering medication and had a moderate to very high cardiovascular risk. The primary variable was the proportion of patients reaching LDL-C goals established by the Fifth Joint European Task Force guidelines. Secondary analyses used McNemar and χ^2^ tests.

**Results:**

Data from 2703 patients were analyzed; 91.2% had a very high cardiovascular risk and 24.0% had been diagnosed with diabetes mellitus. Overall, 17.4% of patients (95% confidence interval [CI] 15.9–18.8%) achieved LDL-C goals. Investigators estimated this proportion at 21.8% (95% CI 20.3–23.4%). LDL-C goals were achieved by more patients in the primary CVD prevention subgroup than in the secondary CVD prevention subgroup (19.7% vs 16.1%, p = 0.017). Patient-related factors associated with a decreased likelihood of achieving LDL-C goals included having ischemic heart disease or a family history of premature coronary heart disease, forgetting to take hypercholesterolemia treatment or considering it acceptable to miss prescribed doses more than once per week, and dissatisfaction with or concern about lipid-lowering therapy. Overall, 367/593 (61.9%) patients with diabetes mellitus and interpretable HbA1c results achieved HbA1c < 7%.

**Conclusions:**

Hypercholesterolemia management is suboptimal in patients with moderate to very high cardiovascular risk in Russian clinical practice. Substantial opportunity remains to improve treatment target attainment and reduce the risk of cardiovascular complications. Lipid-modifying strategies may need to be intensified to reduce CVD risk in this setting.

*Trial registration* ClinicalTrials.gov: NCT02230241 (registered 26 August 2014)

**Electronic supplementary material:**

The online version of this article (10.1186/s12933-017-0641-4) contains supplementary material, which is available to authorized users.

## Background

Cardiovascular disease (CVD) is the leading cause of death globally and imposes a substantial health-economic burden [1]. Elevated levels of total serum cholesterol (TC) and low-density lipoprotein cholesterol (LDL-C) have been identified as strong contributors to CVD mortality [2, 3]. An elevated level of glycosylated hemoglobin (HbA1c) is also an established risk factor for cardiovascular (CV) complications [4–6].

Statins are first-choice medications for reducing hypercholesterolemia [7]. Their use has provided clear benefits in both primary [7] and secondary [8] prevention of CVD. A Cochrane review of trials in which ≤ 10% of participants had a history of CVD showed reductions in all-cause mortality and the risk of CVD events in individuals treated with statins compared with those receiving placebo or usual care for primary CVD prevention, over a minimum follow-up duration of 6 months [7]. Treatment with statins reduced the odds of all-cause death during the observation period by 14% relative to placebo or usual care (odds ratio [OR] 0.86; 95% confidence interval [CI] 0.79–0.94), lowered the risk of CVD (relative risk [RR] 0.75; 95% CI 0.70–0.81), coronary heart disease (CHD) events (RR 0.73; 95% CI 0.67–0.80), and stroke (RR 0.78; 95% CI 0.68–0.89), and decreased the rate of revascularization (RR 0.62; 95% CI 0.54–0.72) [7]. Reductions in TC and LDL-C levels were observed with statins relative to placebo or usual care in trials that reported these outcomes, although there was evidence of heterogeneity of effects, probably owing to differences in the types of statin and dosages used [7]. Statins were also observed to be effective in the prevention of any CV event in a meta-analysis of trials evaluating secondary prevention of CVD (RR vs placebo: 0.81; 95% CI 0.78–0.85) [8].

The Prevalence of Mixed Dyslipidemia and Severe Hypertriglyceridemia in the Russian Population (PROMETHEUS) study found that almost one-third of Russians have hypertriglyceridemia, defined as a triglyceride level ≥ 1.7 mmol/l, and identified correlations between levels of triglycerides and those of TC, LDL-C, high-density lipoprotein cholesterol (HDL-C), and HbA1c [9]. A further observational, cross-sectional study of Russian participants, the Centralized Survey on the Undertreatment of Hypercholesterolemia in Russia (CEPHEUS), was conducted between October 2010 and March 2011, and assessed the use and effectiveness of lipid-lowering drugs in 1000 individuals [10]. The results showed that just under half (48.2%) of the participants who received lipid-lowering therapy achieved LDL-C target levels recommended by the 2007 Fourth Joint European Task Force guidelines [11], and 34.5% achieved LDL-C targets set by the 2007 Russian Society of Cardiology (RSC) guidelines [12]. The proportion of patients achieving these LDL-C levels was lower in individuals receiving lipid-lowering treatment for the primary prevention of CV events than in those receiving it for secondary prevention (27.0% vs 38.2% according to RSC criteria and 35.4% vs 54.5% according to Fourth Joint European Task Force criteria) [10]. Therefore, LDL-C goal attainment appears to be suboptimal in Russia despite the prescription of lipid-lowering treatments, especially among individuals receiving these therapies for primary CVD prevention.

In 2012, after CEPHEUS had been completed [10], the Fifth Joint European Task Force guidelines for CVD prevention were published [13]. These guidelines introduced a more stringent LDL-C target of < 1.8 mmol/l, or ≥ 50% LDL-C reduction when the target could not be reached, for people at very high CV risk [13] (compared with the Fourth Joint European Task Force recommended target of < 2.5, or < 2 mmol/l if feasible, for patients at high CV risk) [11]. In Russia, the medical community received new national guidelines for the prevention and treatment of atherosclerosis in 2009 [14], and an updated version in 2012 [15]. In 2009, the All-Russian Scientific Society of Cardiologists recommended an LDL-C level of < 2.0 mmol/l as ‘optimal’ for patients at both high and very high CV risk [14]. In contrast, in 2012, the renamed RSC proposed an LDL-C target of ≤ 2.5 mmol/l for people at high CV risk and introduced a more stringent target of ≤ 1.8 mmol/l for those at very high CV risk [15]. New risk stratification guidance also defined all patients with CHD (with or without complications) as being at very high CV risk [15]. These changes increased the proportion of patients requiring more stringent targets.

The Centralized Pan-Russian Survey of the Undertreatment of Hypercholesterolemia II (CEPHEUS II) was planned to assess the effects of guideline changes on the attainment of target LDL-C levels in patients with an elevated CV risk in clinical practice in Russia. As CV risk stratification and LDL-C target levels in the 2012 RSC guidelines [15] were based mainly on Fifth Joint European Task Force recommendations [13] with minor differences, we used the latter to define CV risk and treatment goals in this study. We also assessed LDL-C goal attainment among participants prescribed lipid-lowering therapy for primary compared with secondary CVD prevention, predictors of LDL-C goal attainment, and the proportion of patients with diabetes mellitus (DM) who achieved an HbA1c level < 7% (53 mmol/mol).

## Methods

### Study design, aim, and setting

CEPHEUS II was a prospective, multicenter, non-interventional survey of patients with a moderate to very high CV risk receiving lipid-lowering medications (ClinicalTrials.gov identifier: NCT02230241). Its primary aim was to determine the proportion of these patients who reached their LDL-C goal, as defined by the Fifth Joint European Task Force guidelines [13]. The survey was conducted in the Russian Federation between 9 September 2014 and 29 November 2015. In total, 80 sites in different regions of the Russian Federation were selected for this survey; patients were enrolled at 77 outpatient clinics. Data collection took place at a single clinic visit, after which the participation of each patient in the survey was considered to be complete.

Before assessment of the first participant at a site, each investigator completed a questionnaire about their experience and perception of the management of hypercholesterolemia in their patients (see Additional file 1: Figure S1). Before assessment by an investigator, patients completed a questionnaire about their awareness of hypercholesterolemia, their current lipid-lowering treatment schedule, perceptions of treatment, and adherence to treatment. This patient questionnaire has been published in full previously [16].

At the study visit, investigators used patient record forms to collect information regarding each patient’s demographic characteristics, CVD history, known CV risk factors, current lipid-lowering drug therapy, and whether this treatment was for primary or secondary CVD prevention. Physical examinations were conducted to measure the patient’s weight, height, waist circumference, and blood pressure. Fasting blood samples were taken at the study visit and were analyzed to determine levels of TC, LDL-C, HDL-C, glucose, creatinine, HbA1c, hemoglobin, and hematocrit, at a central laboratory (INVITRO, Moscow, Russian Federation). If a participant had not fasted for ≥ 8 h before the visit, fasting blood tests were rescheduled for a different day (within the next 2 days). The patient’s CV risk was categorized according to the Fifth Joint European Task Force guidelines on CVD prevention in clinical practice (Table 1) [13].

**Table 1:** Risk categories and LDL-C goals established by the Fifth Joint European Task Force [13]

Patient characteristics	Risk category	LDL-C goal
CVD diagnosed by invasive or non-invasive testing, peripheral artery disease, any arterial revascularization, ischemic stroke; type 1 or type 2 diabetes mellitus with ≥ 1 CV risk factor and/or target organ damage; GFR < 30 ml/min/1.73 m^2^; and/or calculated SCORE ≥ 10%	Very high	< 1.8 mmol/l (~ 70 mg/dl) or a ≥ 50% LDL-C reduction when the target cannot be reached
Markedly elevated single risk factors such as familial dyslipidemia and severe hypertension; type 1 or type 2 diabetes mellitus without CV risk factors or target organ damage; GFR 30–59 ml/min/1.73 m^2^; and/or calculated SCORE ≥ 5% and < 10%	High	< 2.5 mmol/l (~ 100 mg/dl)
Calculated SCORE ≥ 1% and < 5%	Moderate	< 3.0 mmol/l (~ 115 mg/dl)
Calculated SCORE < 1%	Low	< 3.0 mmol/l (~ 115 mg/dl)

For screening (to exclude potential participants with a low CV risk), use of the CKD-EPI equation was recommended for estimating GFR. The CKD-EPI equation was used to estimate GFR and CV risk for the primary calculation

*CKD*-*EPI* Chronic Kidney Disease Epidemiology Collaboration, *CV* cardiovascular, *CVD* cardiovascular disease, *GFR* glomerular filtration rate, *LDL*-*C* low-density lipoprotein cholesterol, *SCORE* Systematic Coronary Risk Evaluation Project estimation of 10-year risk of fatal cardiovascular disease

The study was conducted in accordance with the principles of the Declaration of Helsinki, the International Conference on Harmonisation Guideline for Good Clinical Practice, and applicable legislation for non-interventional studies. The investigators performed the study in accordance with local regulations and guidelines governing medical practice and ethics. The study was approved by the Independent Interdisciplinary Ethics Committee on Ethical Review for Clinical Studies. All participating patients provided written informed consent.

### Participants

Patients were eligible to participate in this study if they were aged ≥ 18 years, had been receiving lipid-lowering medication for ≥ 90 days with no dose change for ≥ 8 weeks, and had blood tests scheduled to measure levels of TC, LDL-C, HDL-C, glucose, creatinine, HbA1c, hemoglobin, and hematocrit at the screening visit (or if these tests were deemed necessary during the screening visit for reasons unrelated to the study). Individuals were excluded from participation if their cognitive status and/or home environment were judged by the investigator to have potentially compromised adherence to the treatment regimen during the past 8 weeks, if they had a low CV risk, or if they were participating in any other clinical trial.

### Variables

Details of variables assessed during the study are presented in Additional file 2: Table S1. The primary variable was the proportion of patients who reached their LDL-C goal, as defined by the Fifth Joint European Task Force guidelines [13], based on the central laboratory LDL-C test results and stratification of patients by software into three CV risk categories: moderate (LDL-C target: < 3.0 mmol/l [~ 115 mg/dl]), high (LDL-C target: < 2.5 mmol/l [~ 100 mg/dl]), and very high (LDL-C target: < 1.8 mmol/l [~ 70 mg/dl] or a ≥ 50% LDL-C reduction if the target could not be reached) (Table 1). CV risk classification required the calculation of estimated glomerular filtration rate, for which the Chronic Kidney Disease Epidemiology Collaboration (CKD-EPI) equation was used [17].

Secondary variables included the proportion of patients who reached their LDL-C goal among those prescribed lipid-lowering therapy for primary CVD prevention or for secondary CVD prevention, predictors of LDL-C goal attainment, the percentage of CV risk assessments made by physicians that differed from calculated risk classification, and the proportion of patients with DM who achieved an HbA1c level < 7% (53 mmol/mol).

No proactive safety data collection took place. Spontaneously mentioned safety events were reported as required by post-marketing pharmacovigilance regulations.

### Sample size calculation and statistical methods

Based on an estimated 50% of patients achieving their LDL-C goal and a width of the observed two-sided 99% CI of ± 2.5%, a sample size of approximately 2700 patients was considered sufficient to meet the primary objective.

The full analysis set (FAS) included all eligible patients with available laboratory test results who had completed questionnaires for estimation of at least one primary or secondary study variable. This population was used for descriptive analyses of study variables. Missing observations were summarized separately.

Logistic regression analyses were conducted to evaluate the potential association between patient or investigator characteristics and the attainment of LDL-C goals. A multivariate logistic regression model was used to evaluate the association between patient characteristics identified as statistically significant (p ≤ 0.05) in univariate analyses and the attainment of LDL-C goals. A binary categorical attribute, LDL-C within or outside the LDL-C target range according to the Fifth Joint European Task Force guidelines, served as a dependent variable in this model. Model-based point estimates of ORs and corresponding 95% CIs were calculated. Only patients with correctly and fully completed patient record forms and questionnaires were included in the multivariate logistic regression analyses.

A post hoc retrospective analysis was performed to assess changes in levels of LDL-C, HbA1c and creatinine between the start of lipid-lowering therapy (using the last available results before initiation of this treatment) and the time of study enrollment.

Statistical comparisons were made using McNemar’s test or a χ^2^ test. All statistical analyses were carried out at the two-tailed 5% significance level. Data are presented as mean and standard deviation (SD) unless otherwise stated.

## Results

### Patient disposition

In total, 2704 patients were enrolled in the study. One enrolled patient was excluded from the FAS owing to a lack of central laboratory data.

### Patient characteristics

Patient demographics and baseline characteristics are shown in Table 2. The mean (SD) age of the study population was 62.7 (10.0) years and 53.1% of the patients were men. Most of the patients had a history of CVD or had CV risk factors (e.g. arterial hypertension [92.9%], family history of premature CHD [35.6%], DM [24.0%], or tobacco smoking [18.2%]), and 91.2% were categorized as being at very high CV risk. Although having a low CV risk was an exclusion criterion, two patients included in the FAS (0.1%) were in this category. Most patients (83.7%) were overweight or obese. The mean (SD) LDL-C level was 2.76 (1.02) mmol/l.

**Table 2:** Patient demographics and baseline characteristics

Characteristic	Full analysis set (n = 2703)
Age (years)	
< 40	46 (1.7)
40–54	470 (17.4)
55–69	1529 (56.6)
≥ 70	658 (24.3)
Mean ± SD	62.7 ± 10.0
Sex	
Men	1436 (53.1)
Women	1267 (46.9)
Race	
Caucasian	2693 (99.6)
Asian	9 (0.3)
African	1 (0.0)
BMI (kg/m^2^)	
< 25	441 (16.3)
25–29	1133 (41.9)
≥ 30	1129 (41.8)
Mean ± SD	29.5 ± 4.8
Waist circumference (cm)	97.0 ± 12.7
Systolic blood pressure (mmHg)	135.1 ± 16.0
Diastolic blood pressure (mmHg)	82.3 ± 9.5
Total cholesterol (mmol/l)	4.69 ± 1.19
LDL-C (mmol/l)	2.76 ± 1.02
HDL-C (mmol/l)	1.24 ± 0.32
Glucose (mmol/l)	6.16 ± 2.13
Creatinine (µmol/l)	81.29 ± 22.06
HbA1c (%)	5.84 ± 0.97
HbA1c (mmol/mol)^a^	40 ± 11
Hemoglobin (g/dl)	14.19 ± 1.35
Hematocrit (%)	42.17 ± 3.62
eGFR (ml/min/1.73 m^2^)^b^	
< 30	14 (0.5)
30–59	309 (11.4)
≥ 60	2380 (88.1)
CV risk factors	
Arterial hypertension	2511 (92.9)
Family history of premature CHD	963 (35.6)
Previously diagnosed diabetes mellitus	650 (24.0)
Tobacco smoker	491 (18.2)
CVD history	
≥ 1 diagnosis	2254 (83.4)
Myocardial infarction	1122 (41.5)
Angina	1306 (48.3)
Unstable angina	428 (15.8)
Revascularization	1047 (38.7)
Peripheral arterial disease	736 (27.2)
Ischemic stroke	222 (8.2)
Stroke of unknown etiology	46 (1.7)
Other significant concomitant diseases	
Microalbuminuria	248 (9.2)
Macroalbuminuria	50 (1.8)
Diabetic retinopathy	222 (8.2)
Diabetic neuropathy	221 (8.2)
CV risk category^b^	
Low	2 (0.1)
Moderate	86 (3.2)
High	151 (5.6)
Very high	2464 (91.2)

Numbers are n (%) or mean ± SD. Data were available for all 2703 patients in the full analysis set, except for waist circumference (n = 2702) and levels of glucose (n = 2687), HbA1c (n = 2594), hemoglobin, and hematocrit (both n = 2579)

*BMI* body mass index, *CHD* coronary heart disease, *CV* cardiovascular, *CVD* cardiovascular disease, *eGFR* estimated glomerular filtration rate, *HbA1c* glycosylated hemoglobin, *HDL*-*C* high-density lipoprotein cholesterol, *LDL*-*C* low-density lipoprotein cholesterol, *SD* standard deviation

^a^Calculated, not measured

^b^Calculated using central laboratory data

### Current lipid-lowering therapy

All patients were receiving lipid-lowering therapy, with a median treatment duration of 2 years (range 0–23 years). Overall, 99.7% of patients received statins, either as monotherapy or in combination with other agents (Table 3).

**Table 3:** Lipid-lowering drug treatment

Treatment	Full analysis set (n = 2703)
Current treatment	
Statin monotherapy	2645 (97.9)
Fibrates	7 (0.3)
Ezetimibe	2 (0.1)
Statin + fibrates	22 (0.8)
Statin + ezetimibe	23 (0.9)
Statin + fibrates + ezetimibe	2 (0.1)
Missing	2 (0.1)
Reason for treatment	
Primary prevention	943 (34.9)
Secondary prevention	1760 (65.1)

Numbers are n (%)

Lipid-lowering therapy was prescribed for secondary prevention in 65.1% of patients and for primary CVD prevention in the remaining patients (Table 3). The most frequently prescribed statins were atorvastatin (55.7% of patients; most common dose: 20 mg/day [32.2% of patients]) and rosuvastatin (33.9% of patients; most common dose: 10 mg/day [21.6% of patients]). Simvastatin was prescribed in 9.9% of patients (most common dose: 20 mg/day [6.4%]) and lovastatin in two patients (0.1%; 10 and 20 mg/day, respectively). Fenofibrate was prescribed in 1.1% of patients (160 mg/day in one patient, 145 mg/day in the other 30 patients) and ezetimibe in 1.0% of patients (10 mg/day).

### Changes in LDL-C, HbA1c, and creatinine levels following initiation of lipid-lowering therapy

Data for the retrospective post hoc analysis of changes in levels of LDL-C, HbA1c, and creatinine were available for approximately half of the study participants. A decrease in mean value was observed for each of the three parameters between the start of lipid-lowering therapy (using the last available results before initiation of this therapy) and the time of study enrollment. The mean (SD) LDL-C concentration decreased from 4.42 (± 1.464) mmol/l to 2.76 (± 1.016) mmol/l, with a median change of − 1.52 mmol/l (− 37.3%) the mean HbA1c level decreased from 6.63% (± 1.359%) to 5.84% (± 0.969%), with a median change of − 0.10%, and the mean creatinine concentration decreased from 88.85 (± 21.635)–81.29 (± 22.056) µmol/l, with a median change of − 7.00 µmol/l.

### Attainment of LDL-C and HbA1c goals, and prediction of CV risk

**Fig. 1:**
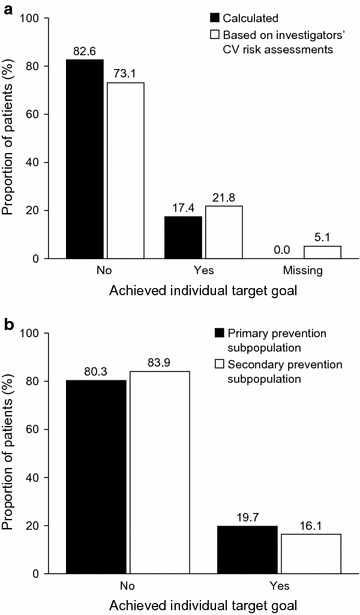
Proportions of patients achieving their low-density lipoprotein cholesterol goal. **a** Calculated proportion and proportion based on investigators’ CV risk assessments for the overall population. **b** Calculated proportion for the primary and secondary prevention subpopulations. Data are for the full analysis set. *CV* cardiovascular

Based on the central laboratory LDL-C test results and CV risk stratification, 17.4% (95% CI 15.9–18.8%) of the study population achieved their LDL-C goal, as defined by the Fifth Joint European Task Force guidelines (Fig. 1a). Differences between patients’ LDL-C level and individual target level in participants at very high CV risk who did not achieve their LDL-C goal are shown in Additional file 3: Figure S2. The difference was ≤ 1 mmol/l in 55.0% of these patients.

According to investigators’ estimations, the proportion of patients achieving their LDL-C goal was 21.8% (95% CI 20.3–23.4%) (Fig. 1a). Differences between the investigators’ CV risk assessments and the primary CV risk calculation using central laboratory data were recorded for 21.3% of patients (p < 0.001 for the differences between assessed CV risks; McNemar’s test). CV risk was underestimated by investigators in 19.4% of patients and overestimated in 1.9% (Table 4). LDL-C goals were achieved by a significantly higher proportion of patients in the primary CVD prevention subgroup than in the secondary CVD prevention subgroup (19.7% vs 16.1%, p = 0.017 [χ^2^ test]; Fig. 1b).

**Table 4:** Calculated CV risks and investigators’ CV risk assessments

CV risk category	Primary calculation using central laboratory data (n = 2703)	Investigators’ assessments (n = 2703)
Low	2 (0.1)	34 (1.3)
Moderate	86 (3.2)	283 (10.5)
High	151 (5.6)	295 (10.9)
Very high	2464 (91.2)	1954 (72.3)
Missing	0 (0.0)	137 (5.1)
Difference between calculated CV risks and investigators’ CV risk assessments (n = 2566)*		
Underestimation by investigators	497 (19.4)	
Same estimation	2020 (78.7)	
Overestimation by investigators	49 (1.9)	

Numbers are n (%) and are for the full analysis set

*CV* cardiovascular

* p < 0.001 for the differences between assessed CV risks (McNemar’s test)

Overall, 367 of 593 (61.9%) patients with DM and interpretable HbA1c results achieved an HbA1c level < 7% (53 mmol/mol).

### Patient-reported variables

Most patients reported awareness of a problem with their LDL-C level or HDL-C level (88.2 and 79.2%, respectively). Overall, 67.9% of patients recalled being informed about their target cholesterol level by their physician; 33.0% of patients in the FAS reported being sure that they had achieved their LDL-C target, while 45.1% were unsure. In total, 89.8% of patients reported taking their prescribed lipid-lowering medications daily, and 84.9% stated that they were satisfied with the way their high cholesterol had been treated. Feelings of concern, frustration, disappointment, or confusion about their treatment were reported by 27.0, 12.1, 5.2, and 4.5% of patients, respectively.

### Investigator-related variables

Of 112 investigators, 96 (85.7%) specialized in cardiology, 16 (14.3%) specialized in internal medicine (‘general practitioner’), three (2.7%) specialized in endocrinology, and six (5.4%) had another specialty (more than one specialty could be indicated). The investigators reported setting individual cholesterol goals for 87.9% of their patients, with most using levels of LDL-C, TC, triglycerides, and HDL-C to determine these goals (96.4, 83.9, 70.5, and 57.1% of investigators, respectively). All investigators had used guidelines to help to establish cholesterol targets. The most frequently used guidelines were the ‘Joint European guidelines (SCORE)’ (Systematic Coronary Risk Evaluation Project estimation of 10-year risk of fatal CVD scale; 78.6% of investigators), ‘National (All-Russian Scientific Cardiology Society) guidelines’ (RSC guidelines; 73.2% of investigators), and ‘NCEP ATP III guidelines (Framingham)’ (National Cholesterol Education Program Adult Treatment Panel III guidelines; Framingham scale; 24.1% of investigators).

### Predictors of LDL-C goal attainment

The attainment of LDL-C goals was not significantly associated with age, sex, body mass index (BMI), waist circumference, smoking status, the presence of DM, or the presence of arterial hypertension (see Additional file 2: Table S2).

Ischemic heart disease and a family history of premature CHD were identified as significant negative predictors of LDL-C goal achievement by multivariate regression analyses. Patients who may have forgotten to take hypercholesterolemia treatment or thought it acceptable to miss prescribed doses more than once per week and those who were dissatisfied with or concerned about their lipid-lowering therapy also had a significantly decreased likelihood of achieving their LDL-C goal (see Additional file 2: Table S2).

Investigator-related factors (such as sex, age, professional experience, specialty, and guidelines mainly used to determine individual target cholesterol level) did not appear to affect the achievement of LDL-C goals (see Additional file 2: Table S3).

### Safety

No adverse drug reactions were registered during the study.

## Discussion

CEPHEUS II was a non-interventional study of clinical practice in Russia, evaluating LDL-C goal attainment in patients with a moderate to very high CV risk who were receiving lipid-lowering medications. Although all patients took lipid-lowering medications, < 20% attained their LDL-C goal, as defined by the Fifth Joint European Task Force guidelines [13]. This proportion is substantially lower than the ~ 50% who achieved their LDL-C target according to Fourth Joint European Task Force guidelines (2007) in CEPHEUS [10]. This finding could be partly attributable to changes in treatment guidelines since 2007 regarding treatment goals and CV risk stratification.

Results from both CEPHEUS [10] and the present CEPHEUS II study have highlighted the suboptimal management of hypercholesterolemia in Russian clinical practice. A potential explanation for suboptimal LDL-C control is that physicians underestimate their patients’ CV risk categories, leading to the choice of an insufficiently strict LDL-C target and overestimation of LDL-C goal attainment, resulting in subsequent undertreatment. In this study, investigators underestimated the CV risk in 19.4% of their patients. Furthermore, investigators overestimated LDL-C goal attainment, reporting that 21.8% of patients achieved their LDL-C goal, whereas the actual proportion who achieved their goal was 17.4%, based on the primary calculation (using central laboratory LDL-C test results and CV risk stratification). Encouragingly, investigators correctly estimated the CV risk in approximately 80% of their patients. Nonetheless, some physicians may benefit from training in CV risk assessment, particularly if they do not specialize in the management of patients at increased CV risk.

Another potential explanation for the low proportion of patients attaining their LDL-C goal is the use of insufficient or inadequate dosages of lipid-lowering therapy. The most commonly used lipid-lowering therapies in the current study were atorvastatin and rosuvastatin, followed by simvastatin. Rosuvastatin was most commonly prescribed at a dose of 10 mg/day, while atorvastatin and simvastatin were most commonly prescribed at doses of 20 mg/day. This moderate-intensity statin therapy reduces LDL-C levels on average by ~ 30–< 50% [18]. High-intensity statin therapy (e.g. rosuvastatin 20 mg/day or atorvastatin 40–80 mg/day) reduces LDL-C levels on average by ~ 50% or more and is recommended by American College of Cardiology/American Heart Association (ACC/AHA) guidelines to reduce the risk of atherosclerotic CVD in certain high-risk individuals [18]. In the present study, 91.2% of patients were categorized as being at very high CV risk according to the Fifth Joint European Task Force guidelines; an LDL-C goal of < 1.8 mmol/l would be recommended for these individuals, or a ≥ 50% LDL-C reduction if the target could be reached [13]. Taken together, our findings suggest that the suboptimal achievement of LDL-C goals in Russia may be attributable, at least in part, to the use of inadequate doses of statins among patients with a very high CV risk.

Our findings are consistent with results from large-scale, cross-sectional studies conducted in Europe [19–21]. The EURIKA study included 7641 patients from 12 European countries who were aged ≥ 50 years and had at least one major risk factor but not CVD [19]. Of EURIKA participants receiving lipid-lowering therapy, 38.7% of those with a high CV risk and 17.1% of those with a very high CV risk (including patients with DM) had controlled LDL-C levels, defined as < 2.5 and < 1.8 mmol/l for the respective CV risk groups [19]. Among individuals in the high or very high CV risk EURIKA subgroups who had uncontrolled LDL-C levels, only 8–9% were receiving a high-intensity statin (rosuvastatin ≥ 20 mg/day or atorvastatin ≥ 40 mg/day) [19]. The European Action on Secondary and Primary Prevention by Intervention to Reduce Events (EUROASPIRE) IV survey was conducted at 79 centers across 24 European countries [20]. Of 6648 EUROASPIRE IV participants with CHD, only 19.3% had achieved target LDL-C levels of < 1.8 mmol/l at the time of assessment [20]. While most of these patients were receiving statins, < 40% were prescribed a high-intensity statin [20]. The proportion of patients receiving lipid-lowering medications who achieved their LDL-C goals was numerically lower in the Russian EUROASPIRE IV cohort than in the total study population (15.9% vs 21.1%, respectively) [21].

In contrast with findings from CEPHEUS [10], LDL-C goals were achieved more frequently in the primary CVD prevention subgroup than in the secondary CVD prevention subgroup in the present study (primary vs secondary prevention: 19.7% vs 16.1%, p = 0.017). The difference in findings may partly reflect the use of a more stringent LDL-C target for patients with a very high CV risk, including individuals with established CVD who required secondary CVD prevention, in CEPHEUS II than in CEPHEUS [< 1.8 mmol/l or a ≥ 50% LDL-C reduction when the target could not be reached (Fifth Joint European Task Force guidelines) vs < 2.5 mmol/l or < 2 mmol/l if feasible (Fourth Joint European Task Force guidelines)] [10, 11, 13].

Patients had a decreased likelihood of LDL-C goal achievement if they had ischemic heart disease or a family history of premature CHD. Those who did not adhere to hypercholesterolemia treatment or who were dissatisfied with or concerned about their lipid-lowering therapy also had a decreased likelihood of achieving their LDL-C goal. Strategies to address the modifiable factors of patient concerns and non-adherence could be employed to improve LDL-C goal attainment. Some patients reported not remembering being informed of their target cholesterol level by their physician, or feeling confused about their treatment. In addition, many patients were unsure of or overestimated their LDL-C goal attainment status. These findings suggest that hypercholesterolemia management would benefit from improved patient–physician communication.

The achievement of LDL-C goals was not significantly associated with age, sex, BMI, waist circumference, smoking status, the presence of DM, or the presence of arterial hypertension. However, the study may have been underpowered to detect some statistically significant associations. In a report of findings from eight European countries, which included a larger population than the present study (n = 14,478 vs n = 2703), having a normal BMI, not smoking, and not having metabolic syndrome were identified as positive predictors for achieving LDL-C goals recommended by the 2003 European guidelines (Third Joint Task Force) [22].

Overall, 61.9% of CEPHEUS II participants with DM and interpretable HbA1c results achieved an HbA1c level < 7% (53 mmol/mol). This finding is consistent with cross-sectional data from the US National Health and Nutrition Examination Surveys (NHANES; 2007–2010), which examined targets recommended by the American Diabetes Association: 52.5% of individuals with DM achieved an HbA1c level < 7% (53 mmol/mol) and 56.2% achieved an LDL-C level < 2.6 mmol/l (100 mg/dl) [23]. In NHANES, the proportions of patients with DM who achieved HbA1c, blood pressure, and LDL-C targets were significantly higher in 2007–2010 than in 1988–1994, and the prevalence of statin use also increased significantly between these periods (from 4.2 to 51.4%, p < 0.01) [23]. However, DM remains a leading cause of CVD, blindness, kidney failure, and lower-limb amputation in almost all high-income countries [24]. Substantial opportunity remains to improve DM control and to reduce the risk of DM-related complications [25].

Suboptimal treatment of patients with DM and mixed dyslipidemia was also reported in a large-scale, retrospective US database analysis of 53,679 patients [26]. In this study more than 40% of patients with DM and two or three abnormal lipid level measurements received no lipid-modifying therapy during the 6-month follow-up period [26]. It has been estimated that if US Preventive Services Task Force (USPSTF) recommendations from 2016 or ACC/AHA guidelines from 2013 were fully implemented, an additional 15.8 or 24.3% of US adults aged 40–75 years without prior CVD would receive statin treatment, respectively [27]. Many of those who would be recommended to receive statins by the ACC/AHA guidelines, but not by the USPSTF recommendations, would be younger adults and people with DM [27], who could potentially benefit from long-term statin therapy.

Other cross-sectional studies conducted in European settings have highlighted the need to optimize statin therapy in patients with type 2 DM before atherosclerotic CVD develops [28] and to improve control of CV risk factors in patients with DM who have experienced myocardial infarction or stroke [29]. Baseline data from type 2 DM clinical trials have also shown that many people with type 2 DM and CVD or CV risk factors do not receive lipid-lowering therapy and have suboptimal lipid control [30, 31]. Patients with type 2 DM may face a combination of barriers to attaining their treatment goals despite more intensive therapy, particularly if they are obese [30]. In addition to implementing guideline recommendations, new strategies to increase patient motivation and promote healthy behaviors, such as regular participation in physical activity, are required [28, 29, 31, 32]. Several novel therapies for dyslipidemia and its associated risks are in development that may be effective with respect to CVD prevention [33] and could increase the options for bespoke treatment programs in the future.

This study has limitations that should be considered when interpreting the results. First, it was a cross-sectional study, and no prospective, longitudinal assessments were conducted. The sample size was based on the estimation that 50% of patients would achieve their LDL-C goal, whereas the proportion of individuals who were found to have achieved their goal was 17.4%. Therefore, the study may not have been sufficiently powered to detect some positive predictors of LDL-C goal achievement. In addition, the investigator questionnaire and patient questionnaire used were not validated and were employed for exploratory purposes only. Assessment of adherence relied on patient recall rather than more objective measures, such as tablet counts or prescription records. The identification of some negative predictors of LDL-C goal attainment, such as having ischemic heart disease or a family history of premature CHD, may be related to the recommendation for a more stringent LDL-C goal and/or due to higher baseline LDL-C levels in patients with these characteristics. Hence, these variables may not be independent predictors of LDL-C goal attainment. Finally, the study was conducted at outpatient clinics in a single country (the Russian Federation) and only included patients receiving lipid-lowering medication; this may limit its generalizability to other countries and settings.

## Conclusions

The CEPHEUS II survey has provided an overview of hypercholesterolemia treatment in a clinical practice setting, based on a large number of patients. Its findings highlight that the management of hypercholesterolemia is suboptimal in patients with moderate to very high CV risk in Russia. There is considerable scope to improve treatment target attainment in these patients and to reduce the risk of CV complications. Lipid-modifying strategies may need to be intensified to reduce CVD risk in this setting.

## Additional files

**Additional file 1: Figure S1.** Investigator questionnaire. Adapted and reproduced with permission from Chiang CE, et al. J Atheroscler Thromb. 2016;23:567–87. *GP* general practitioner; *HDL-C* high-density lipoprotein cholesterol; *LDL-C* low-density lipoprotein cholesterol; *NCEP ATP* National Cholesterol Education Program Adult Treatment Panel; *SCORE* Systematic Coronary Risk Evaluation Project estimation of 10-year risk of fatal cardiovascular disease.

**Additional file 2: Table S1.** Variables assessed during the study. **Table S2.** Association between patient-related factors and achievement of LDL-C goals. **Table S3.** Lack of association between investigator-related factors and achievement of LDL-C goals.

**Additional file 3: Figure S2.** Differences between measured and target LDL-C levels among patients not achieving their LDL-C target. Data are for non-negative difference values and for patients not achieving their LDL-C goal in the very high cardiovascular risk group. *LDL-C* low-density lipoprotein cholesterol.

## Abbreviations

ACC/AHA: American College of Cardiology/American Heart Association
BMI: body mass index
CEPHEUS: Centralized Survey on the Undertreatment of Hypercholesterolemia in Russia
CEPHEUS II: Centralized Pan-Russian Survey of the Undertreatment of Hypercholesterolemia II
CHD: coronary heart disease
CI: confidence interval
CKD-EPI: Chronic Kidney Disease Epidemiology Collaboration
CV: cardiovascular
CVD: cardiovascular disease
DM: diabetes mellitus
EUROASPIRE: European Action on Secondary and Primary Prevention by Intervention to Reduce Events
FAS: full analysis set
HbA1c: glycosylated hemoglobin
HDL-C: high-density lipoprotein cholesterol
LDL-C: low-density lipoprotein cholesterol
NCEP ATP: National Cholesterol Education Program Adult Treatment Panel
NHANES: National Health and Nutrition Examination Surveys
OR: odds ratio
RR: relative risk
RSC: Russian Society of Cardiology
SCORE: Systematic Coronary Risk Evaluation Project estimation of 10-year risk of fatal cardiovascular disease
SD: standard deviation
TC: total cholesterol
USPSTF: US Preventive Services Task Force
