# Supplementary material for: Suboptimal control of lipid levels: results from the non-interventional Centralized Pan-Russian Survey of the Undertreatment of Hypercholesterolemia II (CEPHEUS II)
Source: Cardiovasc Diabetol. 2017 Dec 16;16:158. doi: 10.1186/s12933-017-0641-4 (PMC5732396; doi:10.1186/s12933-017-0641-4)
Supplement: Supplementary file 2 — Additional file 2: Table S1. Variables assessed during the study. Table S2. Association between patient-related factors and achievement of LDL-C goals. Table S3. Lack of association between investigator-related factors and achievement of LDL-C goals. [file 12933_2017_641_MOESM2_ESM.docx]

Additional material

**Table S1 Variables assessed during the study**

| Variable | Information about assessments undertaken |
| --- | --- |
| Primary variable |  |
| Proportion of patients who reached their LDL-C goal as defined by the Fifth Joint European Task Force guidelines [2] | - Based on fasting blood LDL-C results from a central laboratory and stratification of the patients by software into three CV risk categories (moderate, high, and very high) with different LDL-C goals (Table 1) |
| Secondary variables |  |
| Proportion of patients who reached their LDL-C goal as defined by the Fifth Joint European Task Force guidelines [2] among those prescribed lipid-lowering therapy for primary CVD prevention or for secondary CVD prevention | - As for the primary variable - Calculated for the primary prevention and secondary prevention subpopulations, defined according to information on the indication for lipid-lowering therapy provided at the study visit |
| Predictors of LDL-C goal attainment | - Logistic regression analyses were conducted to evaluate the association between patient or investigator characteristics and attainment of LDL-C goals - A multivariate logistic regression model was developed to evaluate the association between patient characteristics and attainment of LDL-C goals. A binary categorical attribute, LDL-C within/outside of the LDL-C target range according to the guidelines of the Fifth Joint European Task Force, served as a dependent variable in this model. Associations investigated in the multivariate logistic regression analyses were identified by significant univariate analyses (p ≤ 0.05) - Only patients with correctly and fully completed patient record forms and questionnaires were included in these analyses |
| Percentage of incorrect CV risk assessments made by physicians | - CV risk assessments were scored as follows: low risk = 1; moderate risk = 2; high risk = 3; very high risk = 4 - Differences between the calculated CV risks and investigators’ CV risk assessments were quantified. A difference of zero indicated that the calculated CV risk and the investigator’s CV risk assessment were the same. A negative value indicated underestimation of CV risk by the investigator, and a positive value indicated overestimation of CV risk by the investigator - The proportion of discordant estimates (both underestimations and overestimations made by investigators) was calculated |
| Proportion of patients with diabetes mellitus who achieved HbA1c < 7% (53 mmol/mol) | - Based on fasting blood HbA1c results from a central laboratory - Calculated among enrolled patients with interpretable HbA1c levels with previously diagnosed diabetes mellitus or whose central laboratory test results indicated HbA1c ≥ 6.5% (48 mmol/mol) or FPG ≥ 7.0 mmol/l (126 mg/dl) - HbA1c values were considered interpretable when both hemoglobin and hematocrit values were within the central laboratory’s normal ranges, and the patient (in the investigator’s opinion) had no other red blood cell turnover disorder that could significantly affect HbA1c levels |
| Investigator-related variables | - Before the assessment of the first participant at a site, each investigator completed a questionnaire about their experience and perception of the management of hypercholesterolemia in their patients (Fig. S1) - The questionnaire was also used to record the sex, age, professional experience, and speciality of the investigators |
| Patient-reported variables | - Before assessment by an investigator, participants completed a questionnaire about their awareness of hypercholesterolemia, current lipid-lowering treatment schedule, perceptions of treatment, and adherence to treatment [1] |
| Patient demographics | - Age, sex, and race were recorded at the study visit and summarized descriptively |
| Physical examination | - Height (cm), weight (kg), waist circumference (analyzed overall and separately for male and female patients, cm), body mass index (kg/m^2^), and blood pressure (mmHg; in a sitting position after ≥ 5 min of rest) were recorded at the study visit and summarized descriptively |
| History of CVD | - History of CVD (CHD, peripheral artery disease, and cerebrovascular disease) was recorded at the study visit and summarized descriptively |
| Concomitant diseases | - Information regarding concomitant diseases was taken from medical records or assessed at the study visit if not previously recorded, and summarized descriptively |
| Presence of CVD risk factors | - The presence of CVD risk factors (arterial hypertension, family history of premature CHD, previously diagnosed diabetes mellitus, and tobacco smoking status [all yes/no]) was recorded at the study visit and summarized descriptively - Family history of premature CHD was defined as definite myocardial infarction or sudden death before 55 years of age in the patient’s father or another male first-degree relative, or before 65 years of age in the patient’s mother or another female first-degree relative - Arterial hypertension was defined as blood pressure ≥ 140/≥ 90 mmHg or current use of antihypertensive medication owing to blood pressure ≥ 140/≥ 90 mmHg identified previously |
| Retrospective laboratory measurements | - The last TC and LDL-C (mmol/l, mg/dl), HbA1c (%), and serum creatinine (µmol/l, mg/dl) values before the initiation of the drug therapy were identified and summarized descriptively |
| Duration of lipid-lowering therapy | - Calculated based on the year of drug initiation, if known |
| Current lipid-lowering medications | - Current lipid-lowering medications and their prescribed daily doses were recorded at the study visit and summarized descriptively |
| Indication for lipid-lowering therapy | - The reason for lipid-lowering therapy (primary or secondary prevention) was recorded at the study visit |
| Laboratory measurements | - Fasting blood samples were taken at the study visit and analyzed for levels of TC, LDL-C, HDL-C, glucose, creatinine, HbA1c, hemoglobin, and hematocrit at a central laboratory in the Russian Federation - If a participant had not been fasting for at least 8 h before the visit, the fasting blood tests were rescheduled for a different day (within the next 2 days) |
| eGFR | - For screening (to exclude potential participants with a low CV risk), use of the CKD-EPI equation [3] was recommended for estimating GFR - The CKD-EPI equation was used to estimate GFR and CV risk for the primary calculation |
| CV risk category | - CV risk was assessed according to the Fifth Joint European Task Force guidelines on CVD prevention in clinical practice (Table 1) [2] |
| Safety events | - ADRs had to be reported by the investigator for the purposes of pharmacovigilance in accordance with applicable local regulations - An ADR was defined as the development of an undesirable medical condition or the deterioration of a pre-existing medical condition following or during exposure to a medicinal product, suspected to be causally related to the product - All treatment-associated AEs were reported according to the procedures established by the manufacturers of the prescribed therapies - All serious AEs occurring from the day of signing the informed consent form until the time of blood sampling were reported |

*ADR* adverse drug reaction, *AE* adverse event, *CKD-EPI* Chronic Kidney Disease Epidemiology Collaboration, *CHD* coronary heart disease, *CV* cardiovascular, *CVD* cardiovascular disease, *eGFR* estimated glomerular filtration rate, *FPG* fasting plasma glucose, *GFR* glomerular filtration rate, *HbA1c* glycosylated hemoglobin, *HDL-C* high-density lipoprotein cholesterol, *LDL-C* low-density lipoprotein cholesterol, *TC* total cholesterol

**Table S2 Association between patient-related factors and achievement of LDL-C goals**

| Variable | Odds ratio  (95% CI) for achievement of  LDL-C goal | p value |
| --- | --- | --- |
| No significant association with LDL-C goal achievement^a^ |  |  |
| Age category (years)  < 40 vs ≥ 70  40–54 vs ≥ 70  55–69 vs ≥ 70 | 0.44 (0.15–1.24)  1.01 (0.74–1.38)  0.95 (0.75–1.20) | 0.119  0.948  0.652 |
| Sex (male vs female) | 1.04 (0.85–1.27) | 0.696 |
| BMI category (kg/m^2^)  25–< 30 vs < 25  ≥ 30 vs < 25 | 0.88 (0.66–1.17)  0.90 (0.67–1.19) | 0.379  0.444 |
| Waist circumference (cm) | 1.00 (0.99–1.01) | 0.802 |
| Smoking (no vs yes) | 1.12 (0.86–1.45) | 0.415 |
| Diabetes mellitus (no vs yes) | 1.01 (0.80–1.28) | 0.926 |
| Arterial hypertension (no vs yes) | 0.80 (0.53–1.21) | 0.294 |
| Negative predictors of LDL-C goal achievement^b^ |  |  |
| Ischemic heart disease (no vs yes) | 1.75 (1.39–2.21) | < 0.001 |
| Family history of premature CHD (no vs yes) | 1.34 (1.06–1.70) | 0.015 |
| Concerned about current lipid-lowering therapy (yes vs no) | 0.75 (0.58–0.97) | 0.028 |
| Considered it acceptable to miss taking prescribed drugs more than once per week (vs once a month or less) | 0.35 (0.12–0.97) | 0.044 |
| Positive predictors of LDL-C goal achievement^b^ |  |  |
| Satisfied with current lipid-lowering therapy (yes vs no) | 1.54 (1.03–2.30) | 0.034 |
| Does not forget to take lipid-lowering therapy (vs sometimes forgets) | 2.06 (1.58–2.68) | < 0.001 |

*BMI* body mass index, *CHD* coronary heart disease, *CI* confidence interval, *LDL-C* low-density lipoprotein cholesterol

^a^ Data are from logistic regression analyses

^b^ Data are from multivariate logistic regression analyses of risk factors identified through significant univariate analyses (p ≤ 0.05)

**Table S3 Lack of association between investigator-related factors and achievement of LDL-C goals**

| Variable | Odds ratio  (95% CI) for achievement of  LDL-C goal | p value |
| --- | --- | --- |
| Sex (male vs female) | 0.85 (0.64–1.13) | 0.260 |
| Age (years) | 1.00 (0.99–1.01) | 0.562 |
| Professional experience (years) | 1.00 (0.99–1.01) | 0.581 |
| Speciality (internal medicine vs cardiologist/endocrinologist) | 1.24 (0.81–1.90) | 0.333 |
| Laboratory measures used to set individual cholesterol goals  TC (yes vs no)  LDL-C (yes vs no)  HDL-C (yes vs no)  Triglycerides (yes vs no) | 0.88 (0.65–1.19)  1.34 (0.74–2.42)  1.08 (0.88–1.32)  1.12 (0.89–1.40) | 0.399  0.338  0.476  0.341 |
| Guidelines mainly used  Joint European guidelines (SCORE) vs  All-Russian Scientific Cardiology Society  NCEP ATP III guidelines (Framingham) vs  All-Russian Scientific Cardiology Society | 0.89 (0.72–1.10)  0.74 (0.46–1.18) | 0.291  0.199 |
| Communication of cholesterol goals  Proportion of patients provided with a target cholesterol level expressed as an actual number | 1.003  (0.998–1.009) | 0.264 |
| Lipid parameters used to inform patients  TC (yes vs no)  LDL-C (yes vs no)  HDL-C (yes vs no)  Triglycerides (yes vs no) | 1.08 (0.77–1.51)  0.93 (0.66–1.33)  0.94 (0.76–1.15)  0.86 (0.69–1.08) | 0.669  0.703  0.538  0.192 |
| Frequency of patient follow-up to review cholesterol level  More often than once every 3 months vs once a year  Once every 3 months vs once a year  Once every 6 months vs once a year | 0.60 (0.35–1.02)  0.95 (0.62–1.45)  0.82 (0.53–1.26) | 0.059  0.801  0.363 |

Data are from logistic regression analyses

*CI* confidence interval, *HDL-C* high-density lipoprotein cholesterol, *LDL-C* low-density lipoprotein cholesterol, *NCEP ATP* National Cholesterol Education Program Adult Treatment Panel, *SCORE* Systematic Coronary Risk Evaluation Project estimation of 10-year risk of fatal cardiovascular disease, *TC* total cholesterol

# References

1. Chiang CE, Ferrières J, Gotcheva NN, Raal FJ, Shehab A, Sung J, et al. Suboptimal control of lipid levels: results from 29 countries participating in the Centralized Pan-Regional Surveys on the Undertreatment of Hypercholesterolaemia (CEPHEUS). J Atheroscler Thromb. 2016;23:567–87.

2. Perk J, De Backer G, Gohlke H, Graham I, Reiner Z, Verschuren M, et al. European guidelines on cardiovascular disease prevention in clinical practice (version 2012). The Fifth Joint Task Force of the European Society of Cardiology and Other Societies on Cardiovascular Disease Prevention in Clinical Practice (constituted by representatives of nine societies and by invited experts). Eur Heart J. 2012;33:1635–701.

3. Levey AS, Stevens LA, Schmid CH, Zhang YL, Castro AF 3rd, Feldman HI, et al. A new equation to estimate glomerular filtration rate. Ann Intern Med. 2009;150:604–12.
